# Supplementary material for: High-throughput barcoding of nanoparticles identifies cationic, degradable lipid-like materials for mRNA delivery to the lungs in female preclinical models
Source: Nat Commun. 2024 Feb 29;15:1884. doi: 10.1038/s41467-024-45422-9 (PMC10904786; doi:10.1038/s41467-024-45422-9)
Supplement: Supplementary file 1 — Supplementary Information [file 41467_2024_45422_MOESM1_ESM.pdf]

Supplementary Information for

High-Throughput Barcoding of Nanoparticles Identifies Cationic, Degradable Lipid-Like Materials for mRNA Delivery to the Lungs in Female Preclinical Models

Xue, *et al.*

This PDF file includes:

Figures (S1-S29)
Tables (S1-S3)

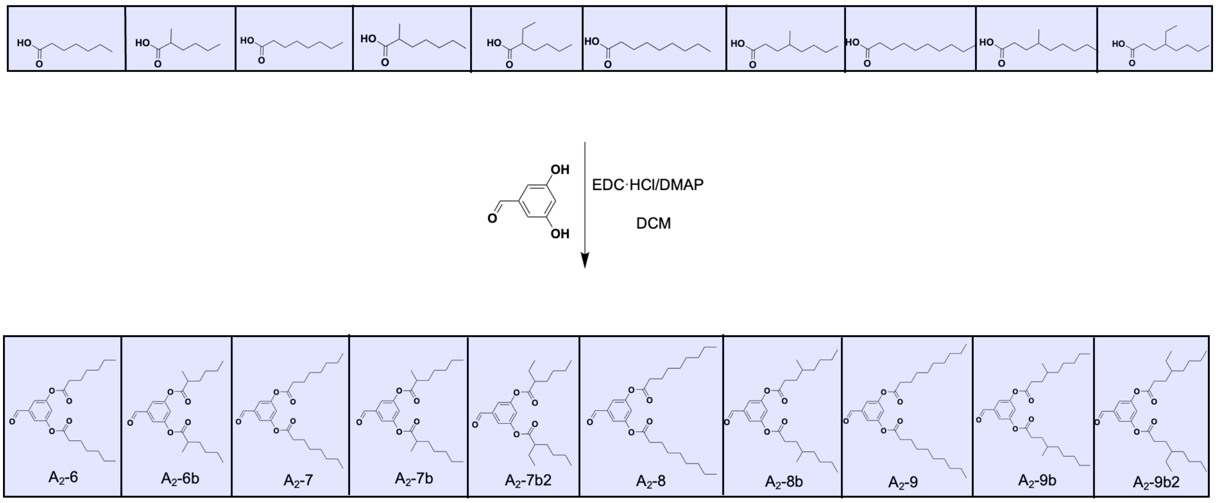

Supplementary Figure 1. Synthetic route of aldehyde di-degradable tails used in this study.

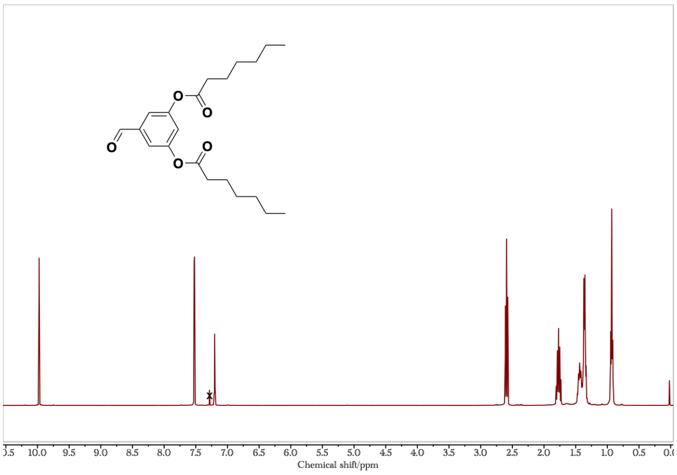

Supplementary Figure 2. ^{1}H NMR spectrum of A_2-6.

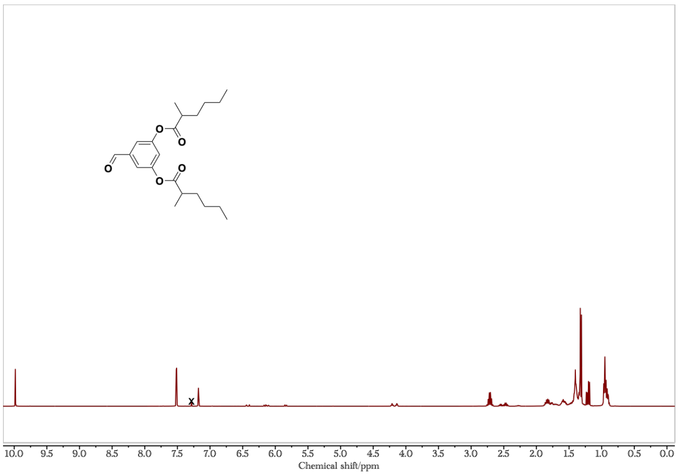

Supplementary Figure 3. ^{1}H NMR spectrum of A_2-6b.

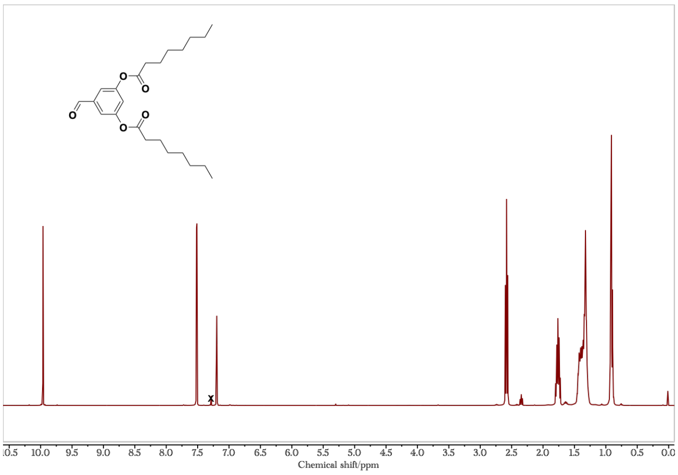

Supplementary Figure 4. ^{1}H NMR spectrum of A_2-7.

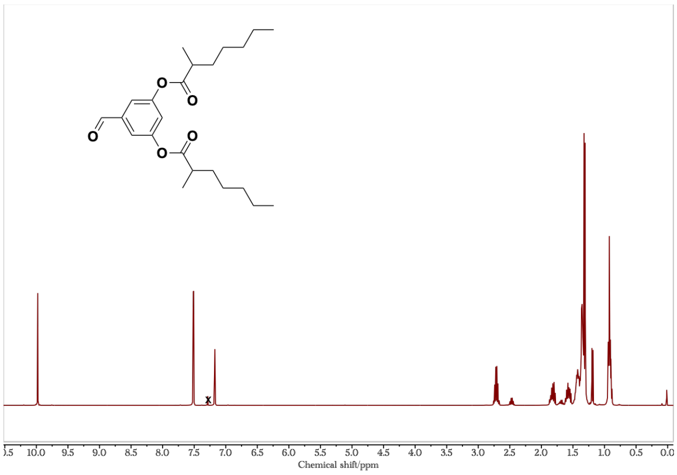

Supplementary Figure 5. ^{1}H NMR spectrum of A_2-7b.

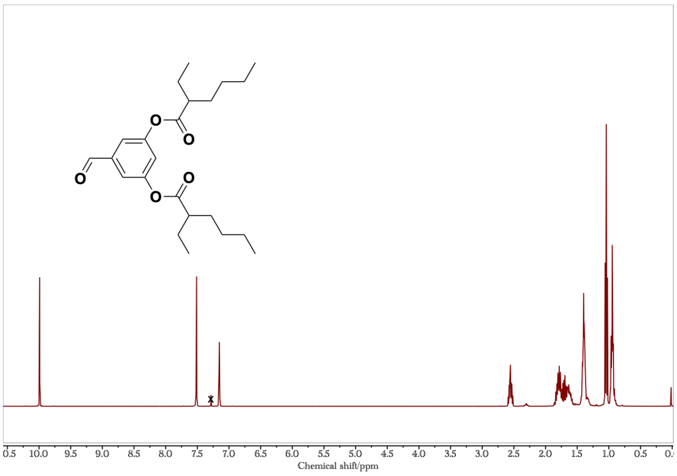

Supplementary Figure 6. ^{1}H NMR spectrum of A_2-7b2.

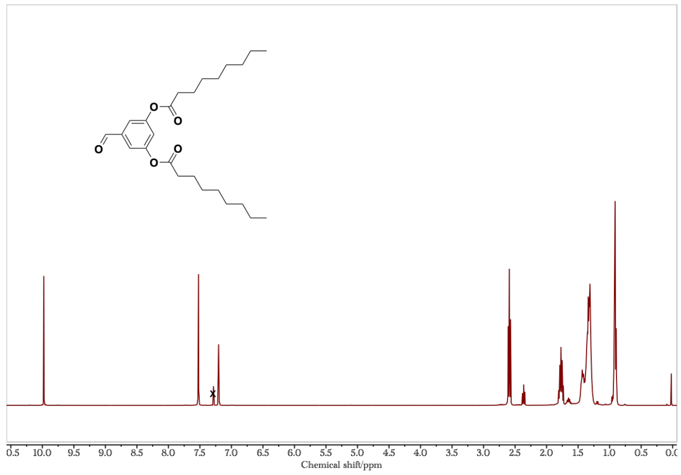

Supplementary Figure 7. ^{1}H NMR spectrum of A_2-8.

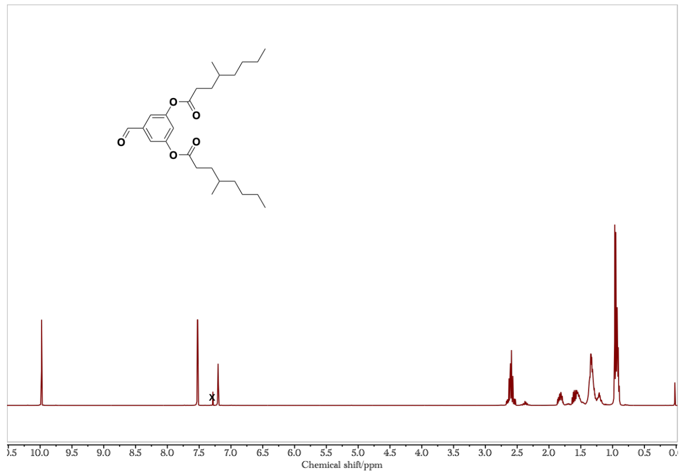

Supplementary Figure 8. ^{1}H NMR spectrum of A_2-8b.

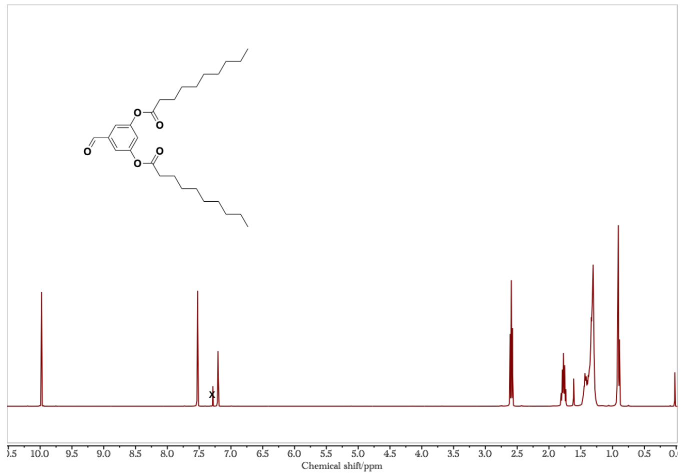

Supplementary Figure 9. ^{1}H NMR spectrum of A_2-9.

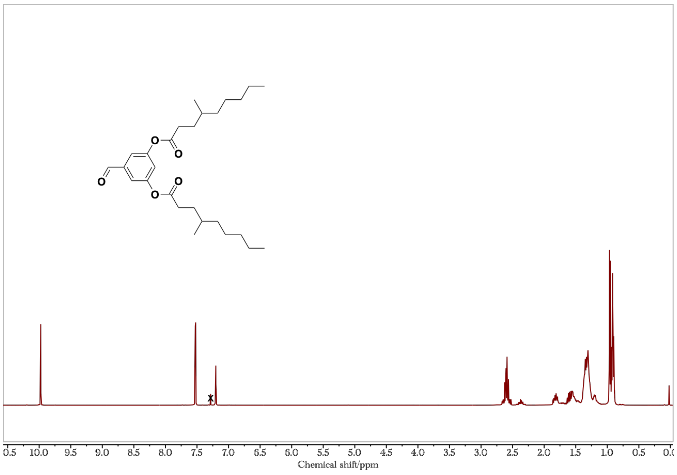

Supplementary Figure 10. ^{1}H NMR spectrum of A_2-9b.

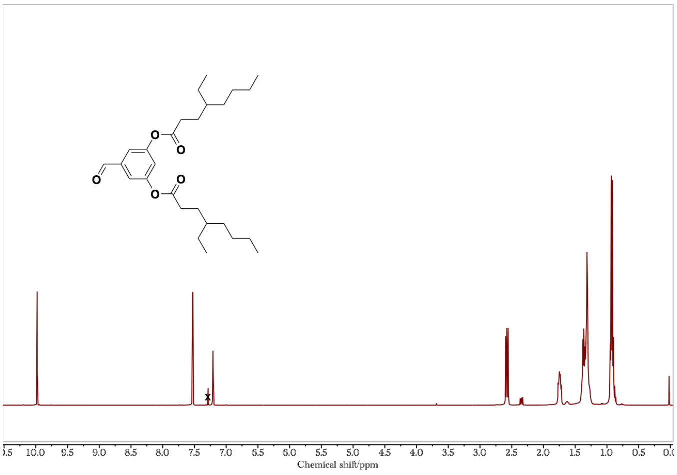

Supplementary Figure 11. ^{1}H NMR spectrum of A_2-9b2.

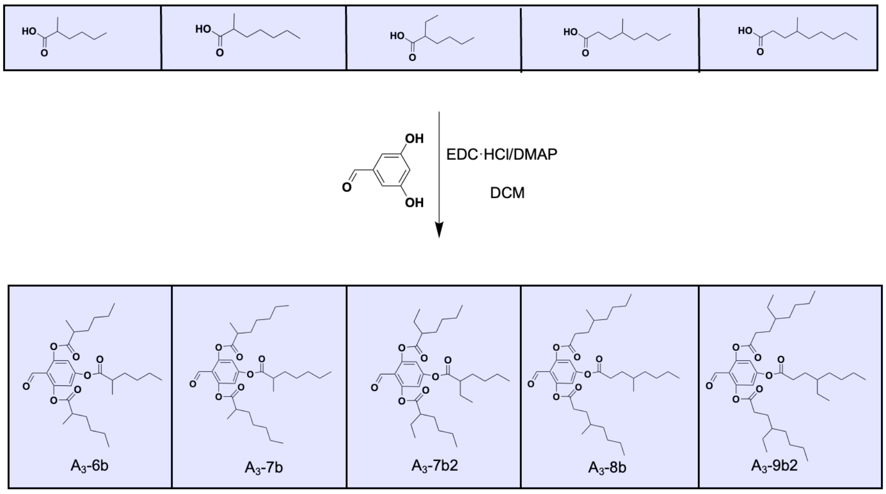

Supplementary Figure 12. Synthetic route of aldehyde tri-degradable tails used in this study.

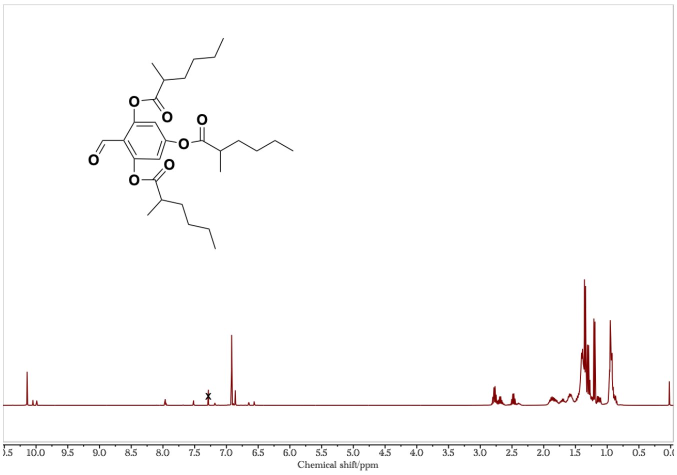

Supplementary Figure 13. ^{1}H NMR spectrum of A$_3$-6b.

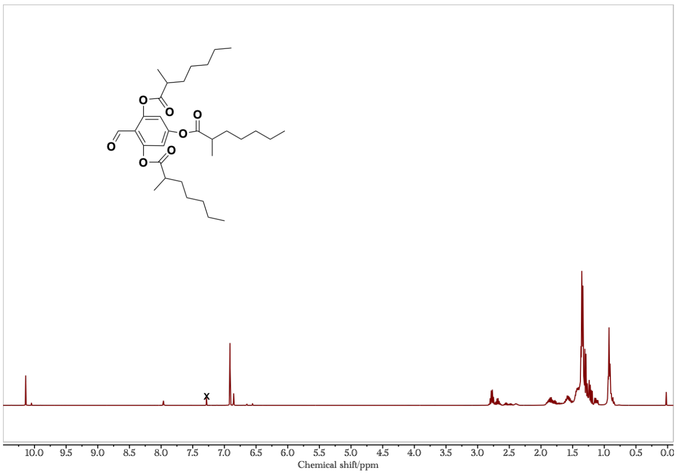

Supplementary Figure 14. ^{1}H NMR spectrum of A$_3$-7b.

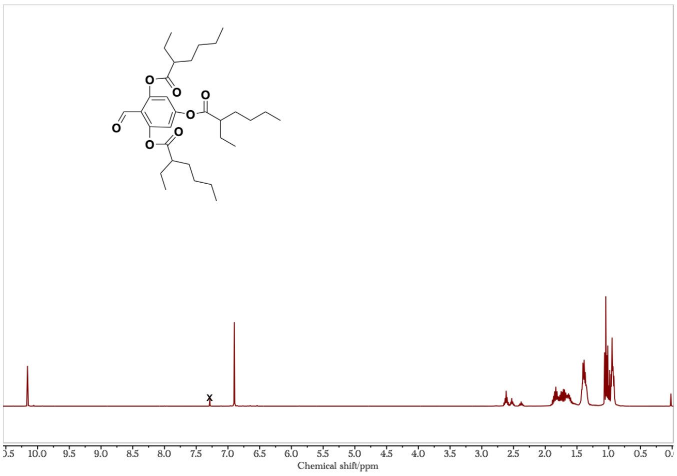

Supplementary Figure 15. ^{1}H NMR spectrum of A$_3$-7b2.

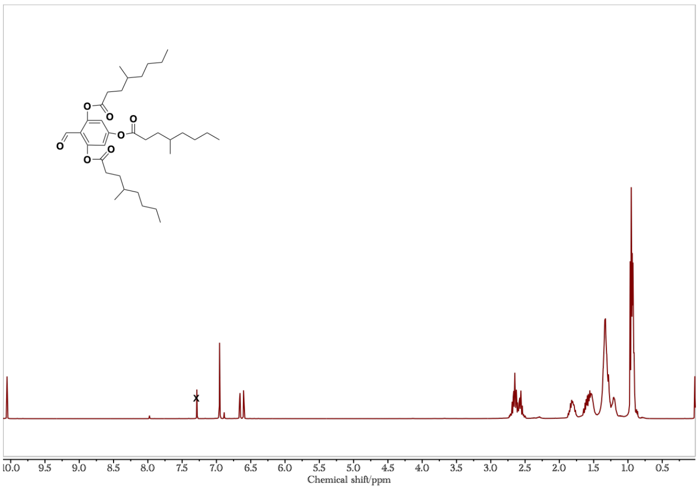

Supplementary Figure 16. ^{1}H NMR spectrum of A_3-8b.

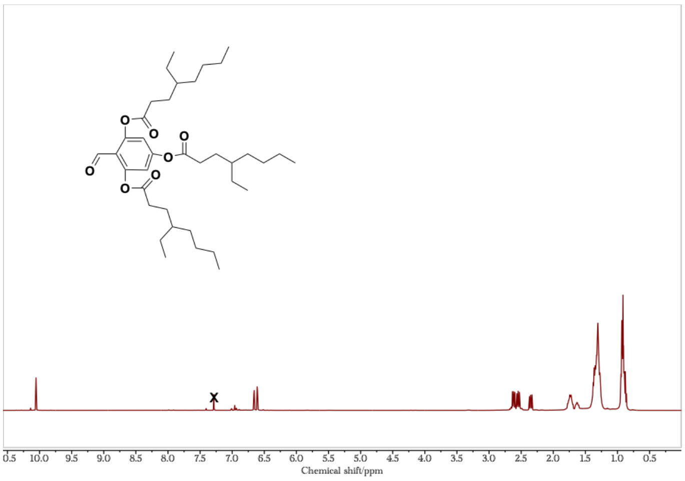

Supplementary Figure 17. ^{1}H NMR spectrum of A_3-9b2.

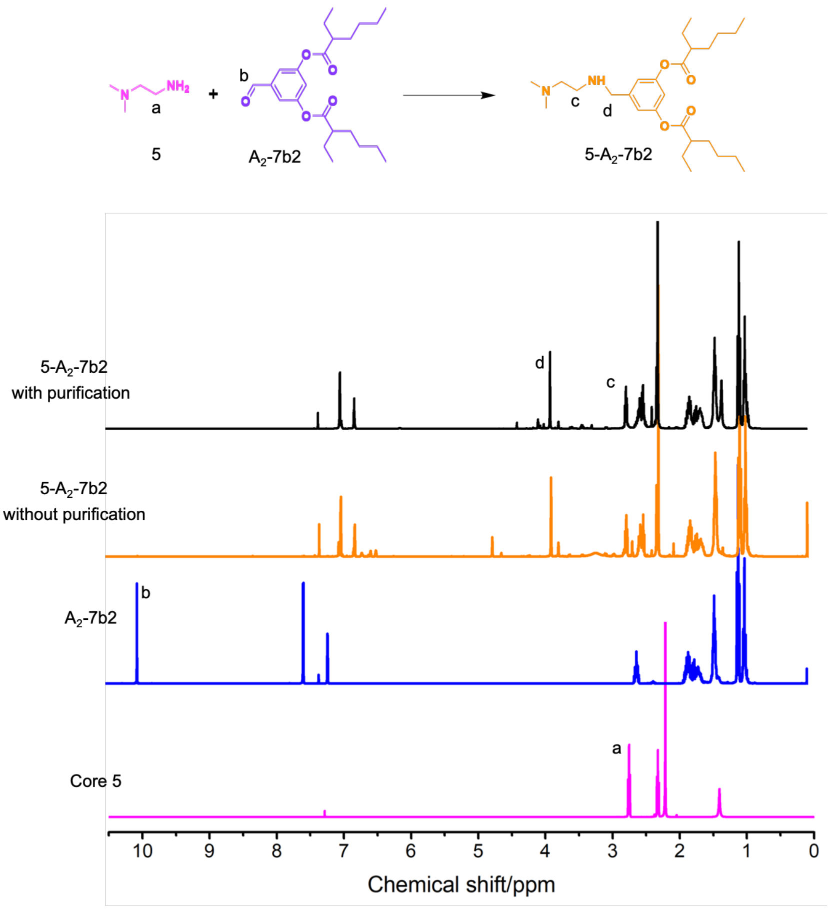

Supplementary Figure 18. Model reaction between amine head 5 and A_2-7b2 and their respective ^{1}H NMR characterizations.

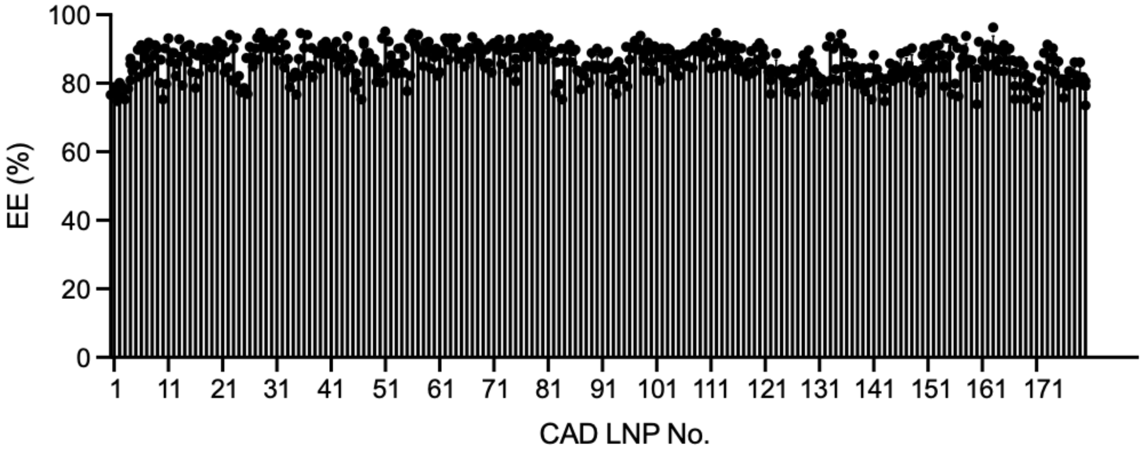

Supplementary Figure 19. mRNA encapsulation efficiency of 180 CAD-LNPs used for *in vitro* screening on HeLa cell (n = 3 replicates). Data are represented as mean ± s.e.m. Source Data are provided in the Source Data File.

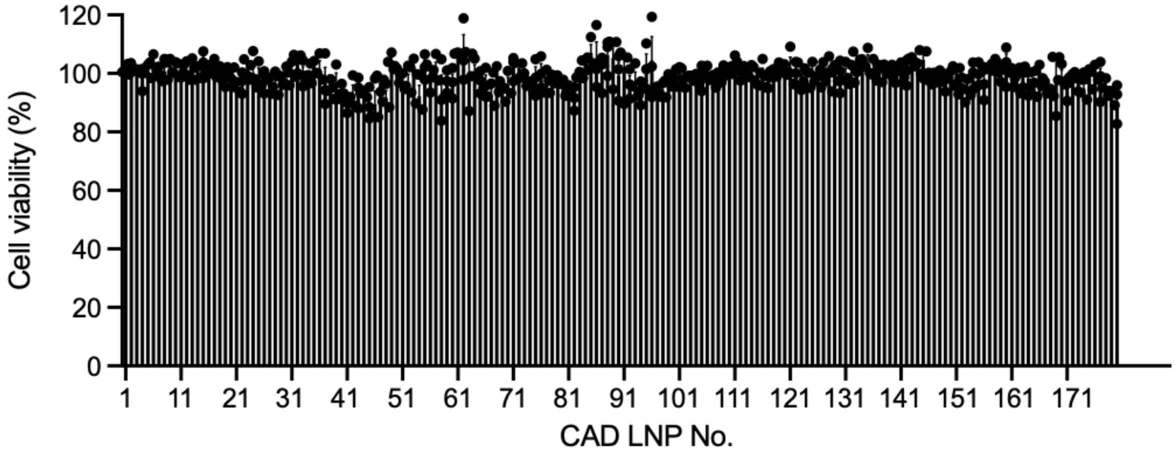

Supplementary Figure 20. Cell viability after 24 h transfection of 180 CAD LNPs in HeLa cells. 5000 cells were plated per well and treated by 10 ng mRNA (n = 3 replicates). Data are represented as mean ± s.e.m. Source Data are provided in the Source Data File.

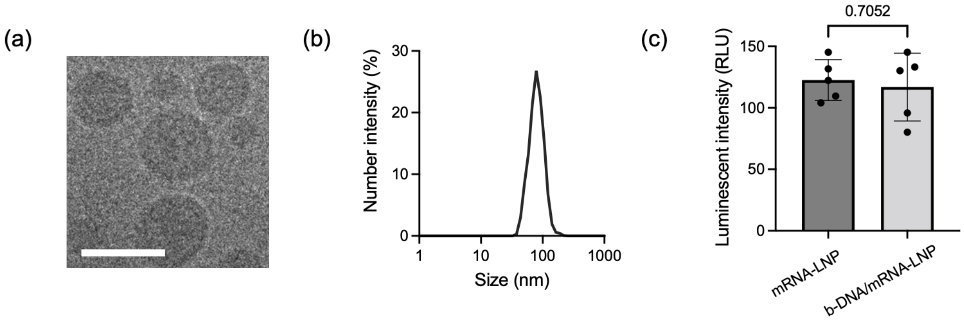

Supplementary Figure 21. (a) Structural morphology of 3-A$_2$-7b LNP encapsulating b-DNA/FLuc (with a weight ratio of 10:1) as visualized by Cryo-TEM. Scale bar: 100 nm. (b) Distribution of hydrodynamic diameter of 3-A$_2$-7b LNP encapsulating b-DNA/FLuc mRNA (with a weight ratio of 10:1) acquired by DLS. (c) Luciferase expression following treatment of HeLa cells with 3-A$_2$-7b LNP encapsulating FLuc mRNA and b-DNA/FLuc mRNA (10 ng luciferase mRNA, n = 5 replicates). Statistical significance in (c) was calculated using Student's t test with unpaired design. *P* > 0.05, not significant (ns). Source Data are provided in the Source Data File.

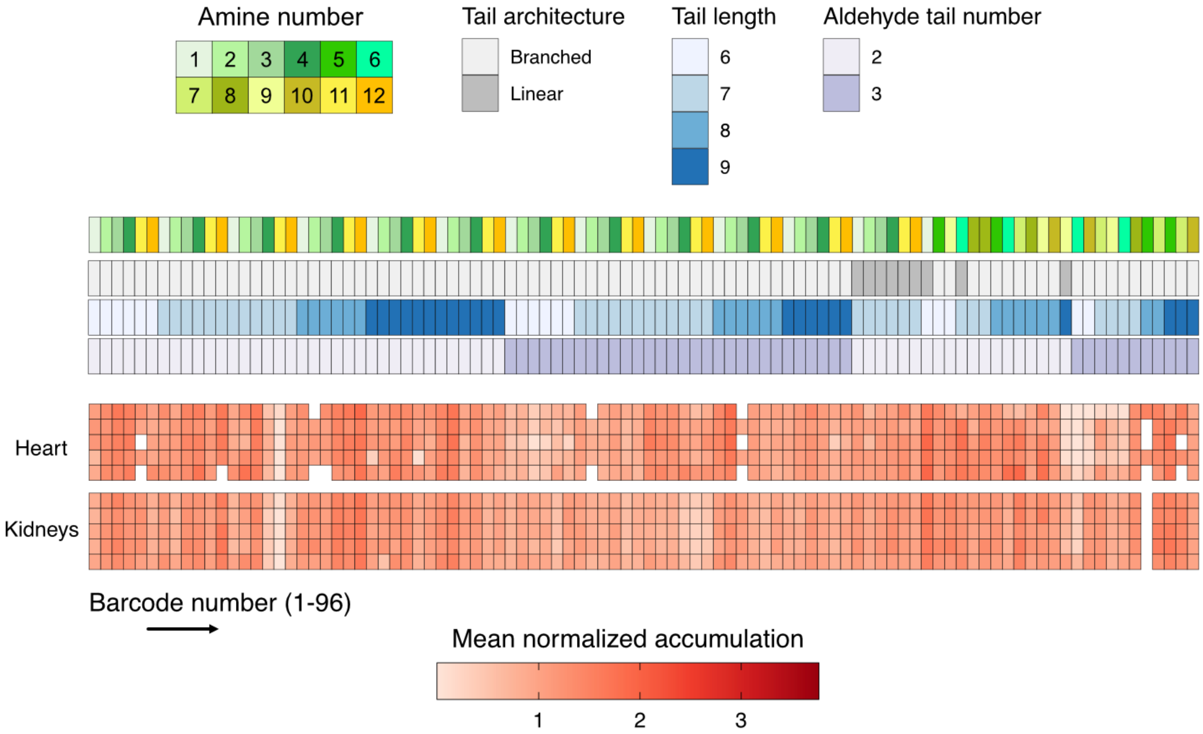

Supplementary Figure 22. Heatmap demonstrates the accumulation of CAD LNPs in the heart and kidneys. Dark clusters represent higher accumulation of a b-DNA in a specific tissue sample. Structure details of CAD lipids in each LNP formulation is described above the heatmap. n = 5 mice.

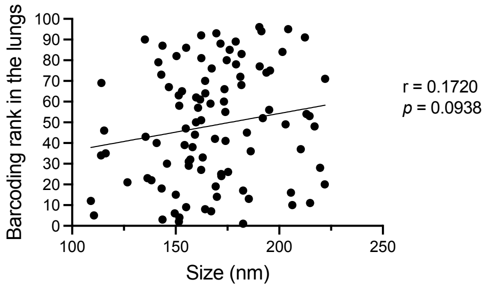

Supplementary Figure 23. Correlation of LNPs size and their respective lung delivery barcoding rank number. Source Data are provided in the Source Data File.

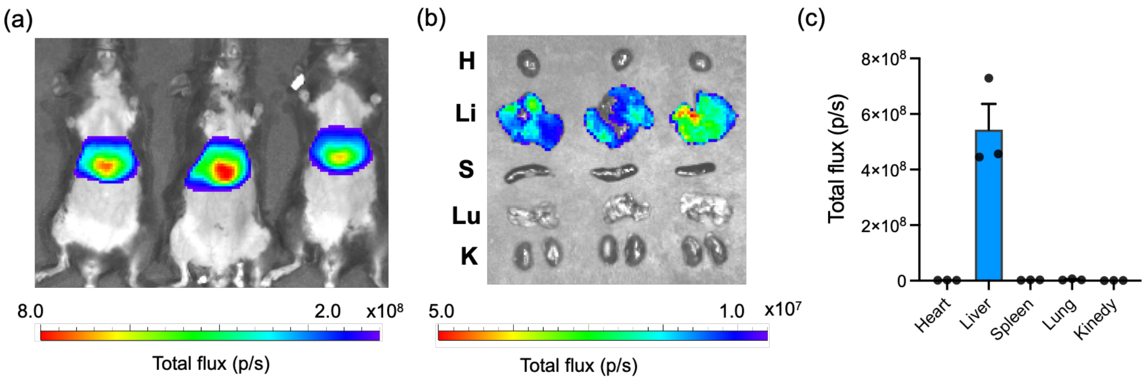

Supplementary Figure 24. *Ex vivo* luminescence intensity of organs in mice after administration with LNP-CAD20 delivering FLuc-mRNA at a dosage of 0.25 mg/kg. (a) Luminescence imaging of the body after LNP treatment. (b) Luminescence imaging of organs from (a). (c) Luminescence quantification of the organs from (b). H: heart; Li: liver; S: spleen; Lu: lung; K: Kidney. Data are represented as mean ± s.e.m. (n =3 mice). Source Data are provided in the Source Data File.

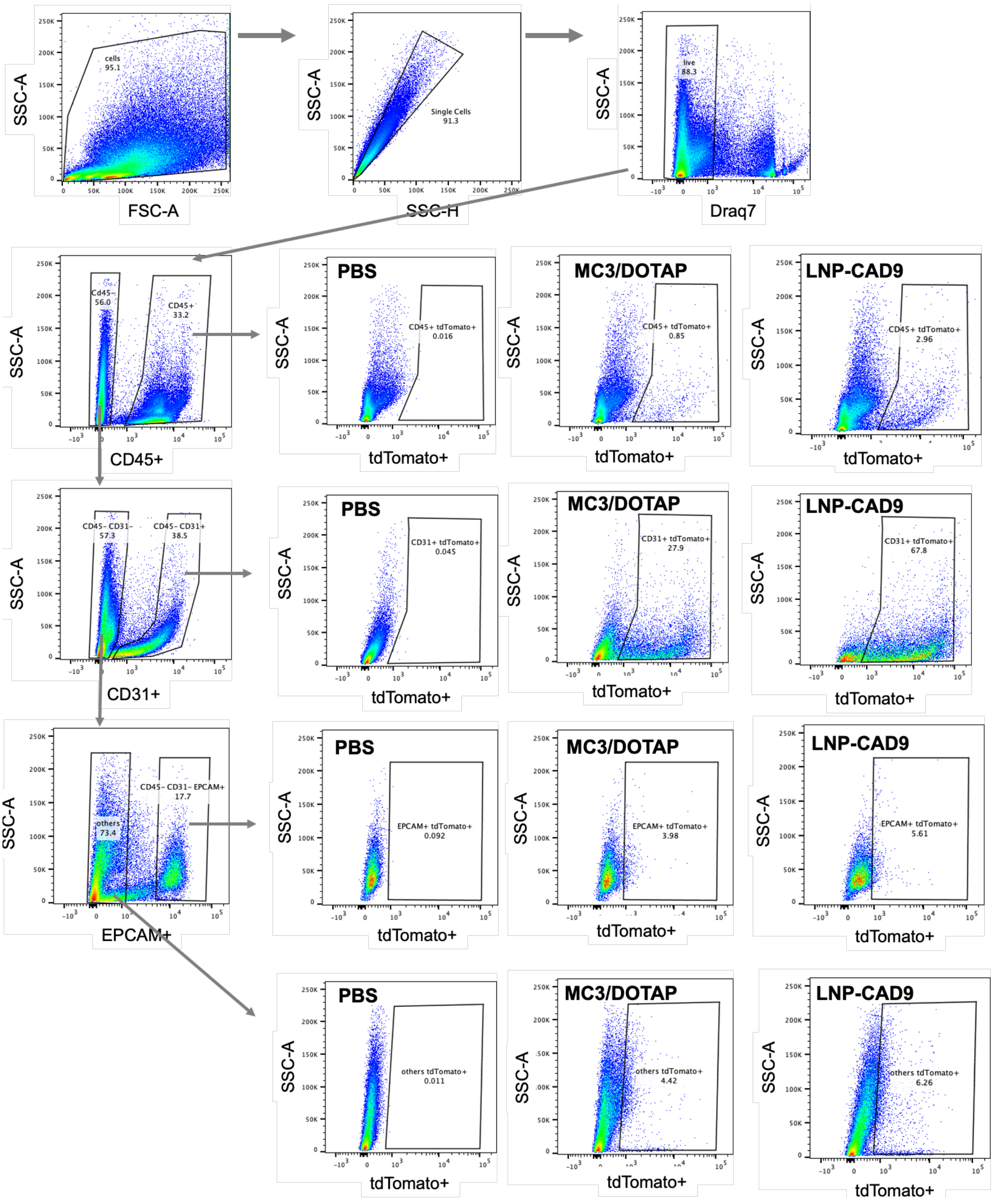

Supplementary Figure 25. Representative gating strategy of diverse tdTomato$^+$ cells in the lungs. Draq7 was used for distinguish live and dead cells. CD45$^+$ antibody was used to stain immune cells, then CD45$^-$/CD31$^+$ was used for lung endothelial cells, CD45$^-$/CD31$^-$/EPCAM$^+$ was used for lung epithelial cells, and the rest CD45$^-$/CD31$^-$/EPCAM$^-$ was stained as others. Ai14 mice was administered with PBS or Cre mRNA LNP (MC3/DOTAP LNP or LNP-CAD9) at a total mRNA dosage of 0.3 mg/kg. The mice were necropsied 3 days post-injection for flow studies. Data are represented as mean ± s.e.m. (n = 4 mice).

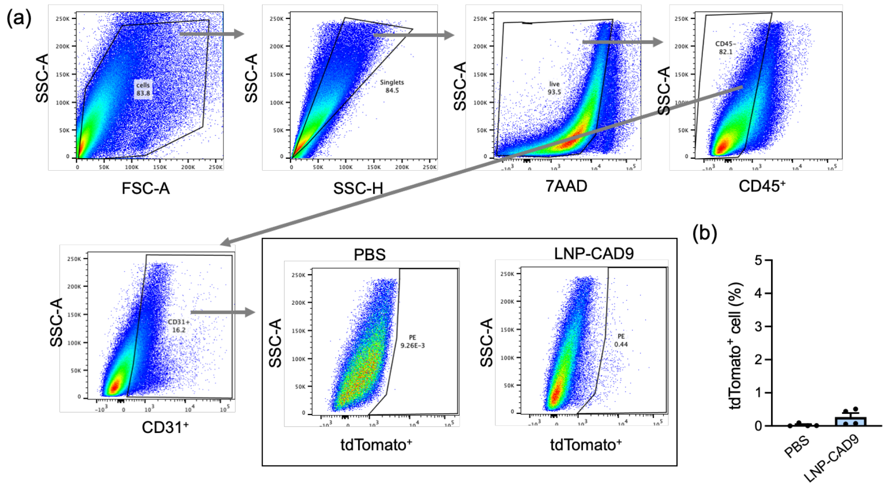

Supplementary Figure 26. (a) Representative gating strategy for tdTomato$^+$ endothelial cells in the liver. 7AAD was used to distinguish live and dead cells. CD45$^+$ antibody was used to stain immune cells, then CD45$^-$/CD31$^+$ was used to identify liver endothelial cells. Ai14 mice was administered with PBS or LNP-CAD9 delivering Cre mRNA at a total dosage of 0.3 mg kg^{-1}. The mice were necropsied 3 days post-injection for flow cytometry studies. (b) Proportion of tdTomato$^+$ endothelial cells in the liver assessed by flow cytometry. Data are presented as ± s.e.m. (n = 4 mice). Source Data are provided in the Source Data File.

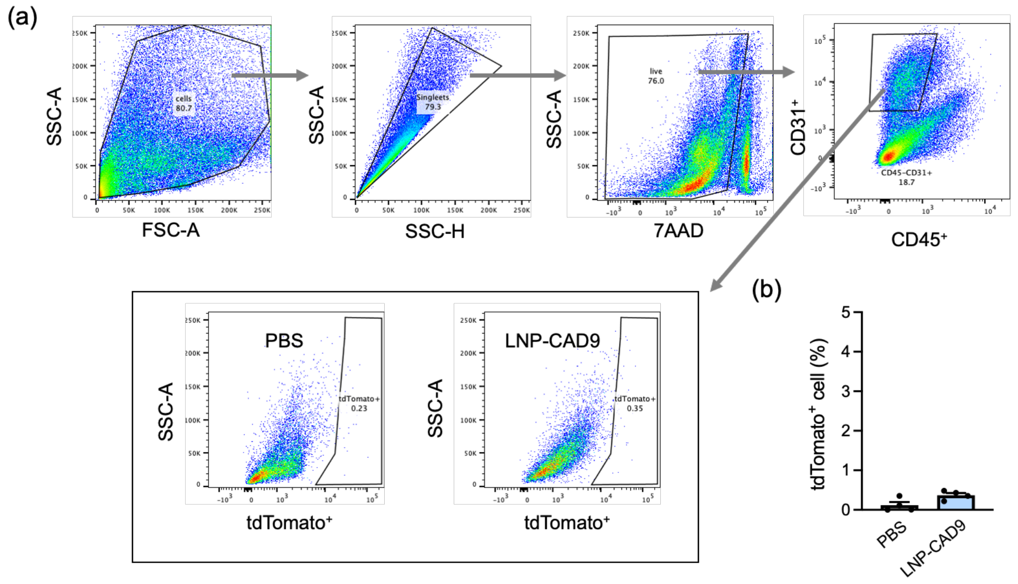

Supplementary Figure 27. (a) Representative gating strategy for tdTomato$^+$ endothelial cells in the heart. 7AAD was used to distinguish live and dead cells. CD45$^+$ antibody was used to stain immune cells, then CD45$^-$/CD31$^+$ was used to identify heart endothelial cells. Ai14 mice was administered with PBS or LNP-CAD9 delivering Cre mRNA at a total dosage of 0.3 mg kg^{-1}. The mice were necropsied 3 days post-injection for flow cytometry studies. (b) Proportion of tdTomato$^+$ endothelial cells in the heart assessed by flow cytometry. Data are presented as ± s.e.m. (n = 4 mice). Source Data are provided in the Source Data File.

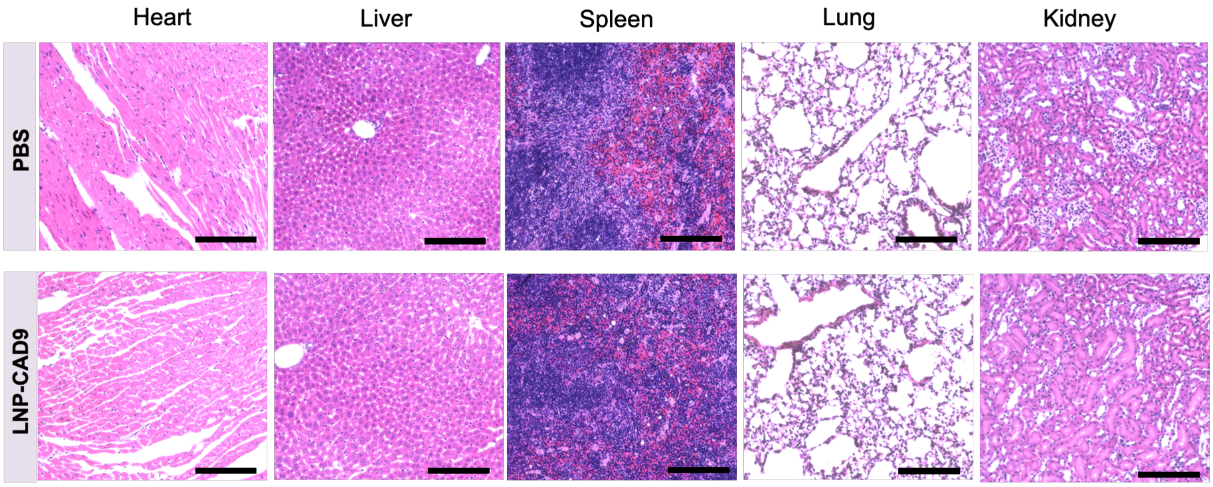

Supplementary Figure 28. Tissue section histology of PBS and LNP-CAD9 treated samples. Mice were administered intravenously at mRNA dosage of 1.0 mg/kg, and PBS treated group was used as the negative control. Tissue sections of heart, liver, spleen, lung, and kidney were acquired 12 h post-injection and prepared for H&E staining. Scale bar = 150 μm.

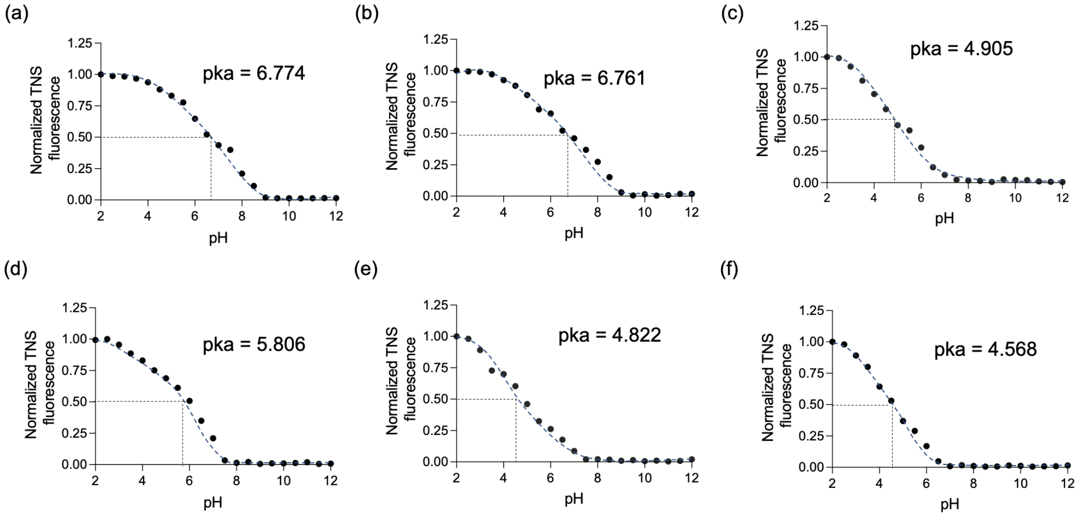

Supplementary Figure 29. Representative TNS assay curves for determining the apparent pKa of LNP-CAD20 (a), LNP-CAD95 (b), LNP-CAD10 (c), LNP-CAD3 (d), LNP-CAD14 (e) and LNP-CAD73 (f). Apparent pKa was defined as the point at which 50% of maximum TNS fluorescence was achieved. Source Data are provided in the Source Data File.

Supplementary Table 1. Particle size, PDI, zeta potential, and encapsulation efficiency (EE) of CAD1-48 based barcode LNPs and its respective CAD lipid information.

Barcode LNP No.	Lipid name	Diamater (nm)	PDI	Zeta potential (mV)	EE (%)	Barcode LNP No.	Lipid name	Diamater (nm)	PDI	Zeta potential (mV)	EE (%)
LNP-CAD1	1-A_2-6b	213.2 ± 3.9	0.40 ± 0.06	1.6 ± 0.8	52.8 ± 2.4	LNP-CAD25	1-A_2-9b	164.2 ± 5.8	0.18 ± 0.08	-1.6 ± 4.4	48.1 ± 0.5
LNP-CAD2	2-A_2-6b	221.9 ± 16.5	0.35 ±0.03	4.2 ± 1.5	69.7 ± 1.8	LNP-CAD26	2-A_2-9b	186.2 ± 4.3	0.26 ± 0.08	5.8 ± 3.9	66.2 ± 3.8
LNP-CAD3	3-A_2-6b	185.3 ± 9.6	0.29 ± 0.05	6.2 ± 0.6	57.4 ± 2.5	LNP-CAD27	3-A_2-9b	169.9 ± 2.8	0.20 ± 0.07	-8.8 ± 2.6	54.9 ± 4.0
LNP-CAD4	4-A_2-6b	205.6 ± 1.9	0.29 ±0.03	7.1 ± 0.9	63.0 ± 2.6	LNP-CAD28	4-A_2-9b	172.1 ± 5.1	0.23 ± 0.07	5.2 ± 1.6	50.9 ± 1.3
LNP-CAD5	11-A_2-6b	219.7 ± 6.2	0.34 ± 0.05	-5.4 ± 0.4	51.5 ± 1.5	LNP-CAD29	11-A_2-9b	153.1 ± 7.8	0.18 ± 0.03	2.9 ± 2.3	48.7 ± 1.4
LNP-CAD6	12-A_2-6b	214.6 ± 3.9	0.31 ± 0.06	6.6 ± 0.5	76.9 ± 1.7	LNP-CAD30	12-A_2-9b	135.1 ± 5.9	0.14 ± 0.04	9.2 ± 3.1	69.4 ± 1.6
LNP-CAD7	1-A_2-7b	182.5 ± 2.8	0.25 ± 0.09	-6.2 ± 0.6	73.4 ± 2.2	LNP-CAD31	1-A_2-9b2	167.1 ± 7.0	0.23 ± 0.05	12.7 ± 2.8	54.9 ± 2.6
LNP-CAD8	2-A_2-7b	217.0 ± 2.7	0.41 ± 0.07	0.1 ± 0.4	77.6 ± 1.9	LNP-CAD32	2-A_2-9b2	164.2 ± 7.6	0.19 ± 0.05	-6.4 ± 2.9	73.2 ± 4.3
LNP-CAD9	3-A_2-7b	150.1 ± 2.9	0.21 ± 0.01	4.5 ± 0.3	71.6 ± 2.1	LNP-CAD33	3-A_2-9b2	157.1 ± 5.7	0.17 ± 0.03	-10.7 ± 3.3	53.1 ± 1.5
LNP-CAD10	4-A_2-7b	214.9 ± 2.0	0.40 ± 0.03	1.1 ± 9.0	78.3 ± 2.2	LNP-CAD34	4-A_2-9b2	171.9 ± 4.0	0.10 ± 0.05	14.7 ± 1.7	64.1 ± 2.1
LNP-CAD11	11-A_2-7b	145.8 ± 2.8	0.15 ± 0.06	-1.6 ± 2.7	73.0 ± 2.6	LNP-CAD35	11-A_2-9b2	166.8 ± 5.2	0.18 ± 0.05	10.4 ± 1.5	57.3 ± 2.4
LNP-CAD12	12-A_2-7b	143.6 ± 6.9	0.22 ± 0.04	-0.4 ± 0.6	74.5 ± 1.9	LNP-CAD36	12-A_2-9b2	160.8 ± 7.6	0.16 ± 0.06	-4.3 ± 5.2	58.4 ± 1.5
LNP-CAD13	1-A_2-7b2	210.3 ± 9.8	0.21 ± 0.05	-1.3 ± 0.8	54.4 ± 4.4	LNP-CAD37	1-A_3-6b	150.3 ± 3.8	0.19 ± 0.06	-9.6 ± 4.7	39.2 ± 1.8
LNP-CAD14	2-A_2-7b2	182.5 ± 8.7	0.24 ± 0.06	-3.0 ± 0.9	59.8 ± 2.9	LNP-CAD38	2-A_3-6b	154.9 ± 5.6	0.13 ± 0.05	1.2 ± 3.7	42.5 ± 1.5
LNP-CAD15	3-A_2-7b2	206.3 ± 6.5	0.31 ± 0.05	1.9 ± 0.2	51.3 ± 1.4	LNP-CAD39	3-A_3-6b	159.3 ± 5.2	0.14 ± 0.07	12.6 ± 3.9	48.8 ± 2.4
LNP-CAD16	4-A_2-7b2	221.1 ± 6.7	0.25 ± 0.07	-8.4 ± 4.8	75.1 ± 1.4	LNP-CAD40	4-A_3-6b	151.4 ± 5.4	0.22 ± 0.07	6.9 ± 2.5	49.6 ± 3.8
LNP-CAD17	11-A_2-7b2	212.3 ± 3.6	0.27 ± 0.04	-0.7 ± 1.6	54.8 ± 2.3	LNP-CAD41	11-A_3-6b	135.4 ± 7.5	0.12 ± 0.05	9.2 ± 1.5	46.8 ± 5.1
LNP-CAD18	12-A_2-7b2	173.2 ± 4.4	0.21 ± 0.06	4.3 ± 0.4	85.6 ± 2.9	LNP-CAD42	12-A_3-6b	156.2 ± 9.1	0.16 ± 0.08	-3.6 ± 1.9	37.0 ± 3.8
LNP-CAD19	1-A_2-8b	136.4 ± 4.9	0.23 ± 0.06	2.8 ± 1.4	84.3 ± 3.1	LNP-CAD43	1-A_3-7b	158.1 ± 10.2	0.15 ± 0.07	6.7 ± 4.0	53.4 ± 3.2
LNP-CAD20	2-A_2-8b	138.3 ± 6.9	0.21 ± 0.12	-0.8 ± 0.6	75.1 ± 1.3	LNP-CAD44	2-A_3-7b	176.0 ± 5.6	0.16 ± 0.04	7.4 ± 1.6	77.9 ± 3.2
LNP-CAD21	3-A_2-8b	116.2 ± 7.6	0.27 ± 0.09	-3.6 ± 3.2	85.7 ± 2.3	LNP-CAD45	3-A_3-7b	167.4 ± 5.1	0.16 ± 0.02	8.9 ± 9.2	60.2 ± 2.8
LNP-CAD22	4-A_2-8b	110.5 ± 9.9	0.22 ± 0.08	2.6 ± 1.4	77.3 ± 2.3	LNP-CAD46	4-A_3-7b	159.8 ± 2.2	0.10 ± 0.04	2.4 ± 0.6	66.7 ± 4.0
LNP-CAD23	11-A_2-8b	149.1 ± 4.7	0.25 ± 0.08	-0.1 ± 1.9	71.9 ± 2.4	LNP-CAD47	11-A_3-7b	143.2 ± 5.3	0.14 ± 0.09	7.0 ± 1.7	45.5 ± 4.4
LNP-CAD24	12-A_2-8b	151.5 ± 4.6	0.23 ± 0.07	3.1 ± 0.6	87.1 ± 2.7	LNP-CAD48	12-A_3-7b	174.6 ± 6.1	0.14 ± 0.03	8.8 ± 2.3	63.3 ± 3.6

Supplementary Table 2. Particle size, PDI, zeta potential, and encapsulation efficiency (EE) of CAD49-96 based barcode LNPs and its respective CAD lipid information.

Barcode LNP No.	Lipid name	Diamater (nm)	PDI	Zeta potential (mV)	EE (%)	Barcode LNP No.	Lipid name	Diamater (nm)	PDI	Zeta potential (mV)	EE (%)
LNP-CAD49	1-A_3-7b2	175.2 ± 3.0	0 26 ± 0.10	1.4 ± 2.5	78.5 ± 4.1	LNP-CAD73	1-A_2-6	203.4 ± 4.9	0.18 ± 0.09	3.8 ± 1.4	85.2 ± 4.9
LNP-CAD50	2-A_3-7b2	163.1 ± 6.5	0.19 ± 0.05	-7.2 ± 2.9	78.8 ± 2.7	LNP-CAD74	5-A_2-6b	174.0 ± 5.5	0.23 ± 0.05	7.9 ± 1.3	81.2 ± 2.7
LNP-CAD51	3-A_3-7b2	162.2 ± 2.7	0.23 ± 0.06	6.2 ± 1.6	79.3 ± 1.7	LNP-CAD75	9-A_2-6b	164.2 ± 5.7	0.13 ± 0.09	6.5 ± 0.9	85.1 ± 2.8
LNP-CAD52	4-A_3-7b2	159.9 ± 3.6	0.10 ± 0.06	5.2 ± 2.4	77.9 ± 6.2	LNP-CAD76	6-A_2-7	143.6 ± 8.7	0.09 ± 0.08	3.3 ± 3.8	85.3 ± 4.4
LNP-CAD53	11-A_3-7b2	162.3 ± 3.0	0.13 ± 0.02	6.4 ± 1.2	79.0 ± 3.7	LNP-CAD77	8-A_2-7b	195.5 ± 9.9	0.29 ± 0.07	-9.7 ± 2.2	80.6 ± 2.9
LNP-CAD54	12-A_3-7b2	151.7 ± 4.9	0.21 ± 0.03	-0.5 ± 1.3	79.4 ± 3.1	LNP-CAD78	8-A_2-7b2	179.2 ± 7.4	0.25 ± 0.09	5.8 ± 1.3	80.6 ± 3.1
LNP-CAD55	1-A_3-8b	155.1 ± 5.2	0.16 ± 0.03	-8.2 ± 1.4	57.8 ± 2.7	LNP-CAD79	5-A_2-8b	181.7 ± 4.9	0.24 ± 0.05	1.4 ± 0.6	83.1 ± 3.0
LNP-CAD56	2-A_3-8b	151.7 ± 3.2	0.25 ± 0.08	1.4 ± 0.9	63.9 ± 3.4	LNP-CAD80	6-A_2-8b	141.6 ± 11.1	0.21 ± 0.04	3.9 ± 2.1	82.4 ± 3.3
LNP-CAD57	3-A_3-8b	162.3 ± 2.6	0.25 ± 0.10	4.7 ± 1.7	41.8 ± 2.2	LNP-CAD81	7-A_2-8b	171.4 ± 4.5	0.16 ± 0.04	6.7 ± 2.0	78.5 ± 4.5
LNP-CAD58	4-A_3-8b	140.8 ± 4.5	0.16 ± 0.07	8.6 ± 0.9	40.6 ± 3.4	LNP-CAD82	8-A_2-8b	155.0 ± 6.5	0.15 ± 0.05	1.5 ± 0.7	79.7 ± 3.5
LNP-CAD59	11-A_3-8b	173.5 ± 5.8	0.20 ± 0.05	-5.6 ± 6.3	57.6 ± 3.0	LNP-CAD83	9-A_2-8b	156.3 ± 5.1	0.26 ± 0.11	6.3 ± 0.9	82.4 ± 3.3
LNP-CAD60	12-A_3-8b	146.7 ± 6.5	0.25 ± 0.06	5.1 ± 2.7	59.2 ± 2.9	LNP-CAD84	10-A_2-8b	190.5 ± 4.3	0.30 ± 0.12	4.3 ± 3.7	83.4 ± 3.1
LNP-CAD61	1-A_3-9b2	161.7 ± 6.8	0.22 ± 0.06	7.9 ± 1.9	46.9 ± 2.5	LNP-CAD85	9-A_2-9	191.3 ± 3.4	0.16 ± 0.10	4.8 ± 2.2	84.4 ± 3.6
LNP-CAD62	2-A_3-9b2	168.9 ± 3.1	0.18 ± 0.02	-2.4 ± 0.9	65.6 ± 3.0	LNP-CAD86	6-A_3-6b	190.4 ± 4.1	0.23 ± 0.08	-1.8 ± 0.8	84.2 ± 2.9
LNP-CAD63	3-A_3-9b2	169.2 ± 5.5	0.22 ± 0.07	2.6 ± 1.8	54.3 ± 2.9	LNP-CAD87	10-A_3-6b	204.3 ± 5.3	0.16 ± 0.07	-6.4 ± 0.6	81.8 ± 4.7
LNP-CAD64	4-A_3-9b2	184.3 ± 6.4	0.12 ± 0.04	5.8 ± 3.4	48.0 ± 2.7	LNP-CAD88	7-A_3-7b	169.6 ± 4.5	0.19 ± 0.12	12.8 ± 0.7	86.8 ± 1.9
LNP-CAD65	11-A_3-9b2	173.9 ± 5.3	0.12 ± 0.03	-0.9 ± 1.2	47.1 ± 5.7	LNP-CAD89	9-A_3-7b	201.5 ± 4.4	0.24 ± 0.06	2.3 ± 0.9	82.4 ± 3.3
LNP-CAD66	12-A_3-9b2	154.3 ± 4.8	0.13 ± 0.07	8.7 ± 1.4	37.2 ± 3.6	LNP-CAD90	6-A_3-7b2	162.3 ± 4.1	0.22 ± 0.06	-1.7 ± 0.6	83.2 ± 4.1
LNP-CAD67	1-A_2-7	192.2 ± 5.1	0.08 ± 0.05	-1.2 ± 0.8	77.8 ± 3.5	LNP-CAD91	8-A_3-7b2	143.2 ± 6.9	0.27 ± 0.11	1.5 ± 2.6	75.9 ± 5.4
LNP-CAD68	2-A_2-7	178.9 ± 3.7	0.18 ± 0.04	1.4 ± 1.9	84.0 ± 6.1	LNP-CAD92	5-A_3-8b	126.7 ± 5.9	0.21 ± 0.09	-1.2 ± 2.7	85.5 ± 4.5
LNP-CAD69	3-A_2-7	193.7 ± 5.0	0.07 ± 0.05	4.4 ± 4.0	74.6 ± 3.5	LNP-CAD93	7-A_3-8b	113.9 ± 5.0	0.13 ± 0.05	-3.1 ± 2.1	73.8 ± 4.0
LNP-CAD70	4-A_2-7	181.2 ± 3.9	0.10 ± 0.05	1.5 ± 7.5	82.2 ± 4.3	LNP-CAD94	5-A_3-9b2	115.4 ± 7.1	0.16 ± 0.08	6.7 ± 4.5	75.1 ± 2.6
LNP-CAD71	11-A_2-7	195.1 ± 6.3	0.12 ± 0.04	8.6 ± 15.5	81.0 ± 4.8	LNP-CAD95	7-A_3-9b2	109.2 ± 5.2	0.25 ± 0.05	-3.0 ± 4.1	80.4 ± 4.3
LNP-CAD72	12-A_2-7	181.8 ± 4.8	0.11 ± 0.09	13.2 ± 0.4	79.8 ± 5.1	LNP-CAD96	10-A_3-9b2	114.1 ± 6.7	0.16 ± 0.04	2.1 ± 1.6	84.9 ± 4.8

Supplementary Table 3. Library of full-length reverse primer sequences. Primer sequences differ in the eight-nucleotide organ barcode sequence.

Number	i7	Sequence
1	CTACCAGG	CAAGCAGAAGACGGCATACGAGATCCTGGTAGGTGACTGGAGTTCAGACGTGTGCTCTTCCGATCT
2	CATGCTTA	CAAGCAGAAGACGGCATACGAGATTAAGCATGGTGACTGGAGTTCAGACGTGTGCTCTTCCGATCT
3	GCACATCT	CAAGCAGAAGACGGCATACGAGATAGATGTGCGTGACTGGAGTTCAGACGTGTGCTCTTCCGATCT
4	TGCTCGAC	CAAGCAGAAGACGGCATACGAGATGTCGAGCAGTGACTGGAGTTCAGACGTGTGCTCTTCCGATCT
5	AGCAATTC	CAAGCAGAAGACGGCATACGAGATGAATTGCTGTGACTGGAGTTCAGACGTGTGCTCTTCCGATCT
6	AGTTGCTT	CAAGCAGAAGACGGCATACGAGATAAGCAACTGTGACTGGAGTTCAGACGTGTGCTCTTCCGATCT
7	CCAGTTAG	CAAGCAGAAGACGGCATACGAGATCTAACTGGGTGACTGGAGTTCAGACGTGTGCTCTTCCGATCT
8	TTGAGCCT	CAAGCAGAAGACGGCATACGAGATAGGCTCAAGTGACTGGAGTTCAGACGTGTGCTCTTCCGATCT
9	ACCAACTG	CAAGCAGAAGACGGCATACGAGATCAGTTGGTGTGACTGGAGTTCAGACGTGTGCTCTTCCGATCT
10	GGTCCAGA	CAAGCAGAAGACGGCATACGAGATTCTGGACCGTGACTGGAGTTCAGACGTGTGCTCTTCCGATCT
11	GTATAACA	CAAGCAGAAGACGGCATACGAGATTGTTATACGTGACTGGAGTTCAGACGTGTGCTCTTCCGATCT
12	TTCGCTGA	CAAGCAGAAGACGGCATACGAGATTCAGCGAAGTGACTGGAGTTCAGACGTGTGCTCTTCCGATCT
13	AACTTGAC	CAAGCAGAAGACGGCATACGAGATGTCAAGTTGTGACTGGAGTTCAGACGTGTGCTCTTCCGATCT
14	CACATCCT	CAAGCAGAAGACGGCATACGAGATAGGATGTGGTGACTGGAGTTCAGACGTGTGCTCTTCCGATCT
15	TCGGAATG	CAAGCAGAAGACGGCATACGAGATCATTCCGAGTGACTGGAGTTCAGACGTGTGCTCTTCCGATCT
16	AAGGATGT	CAAGCAGAAGACGGCATACGAGATACATCCTTGTGACTGGAGTTCAGACGTGTGCTCTTCCGATCT
17	GCACACGA	CAAGCAGAAGACGGCATACGAGATTCGTGTGCGTGACTGGAGTTCAGACGTGTGCTCTTCCGATCT
18	TCTGGCGA	CAAGCAGAAGACGGCATACGAGATTCGCCAGAGTGACTGGAGTTCAGACGTGTGCTCTTCCGATCT
19	CATAGCGA	CAAGCAGAAGACGGCATACGAGATTCGCTATGGTGACTGGAGTTCAGACGTGTGCTCTTCCGATCT
20	CAGGAGCC	CAAGCAGAAGACGGCATACGAGATGGCTCCTGGTGACTGGAGTTCAGACGTGTGCTCTTCCGATCT
21	TGTCGGAT	CAAGCAGAAGACGGCATACGAGATATCCGACAGTGACTGGAGTTCAGACGTGTGCTCTTCCGATCT
22	ATTATGTT	CAAGCAGAAGACGGCATACGAGATAACATAATGTGACTGGAGTTCAGACGTGTGCTCTTCCGATCT
23	CCTACCAT	CAAGCAGAAGACGGCATACGAGATATGGTAGGGTGACTGGAGTTCAGACGTGTGCTCTTCCGATCT
24	TACTTAGC	CAAGCAGAAGACGGCATACGAGATGCTAAGTAGTGACTGGAGTTCAGACGTGTGCTCTTCCGATCT
25	GAAGAAGT	CAAGCAGAAGACGGCATACGAGATACTTCTTCGTGACTGGAGTTCAGACGTGTGCTCTTCCGATCT
26	AGGATCTA	CAAGCAGAAGACGGCATACGAGATTAGATCCTGTGACTGGAGTTCAGACGTGTGCTCTTCCGATCT
27	GACAGTAA	CAAGCAGAAGACGGCATACGAGATTTACTGTCGTGACTGGAGTTCAGACGTGTGCTCTTCCGATCT
28	CCTATGCC	CAAGCAGAAGACGGCATACGAGATGGCATAGGGTGACTGGAGTTCAGACGTGTGCTCTTCCGATCT
29	TCGCCTTG	CAAGCAGAAGACGGCATACGAGATCAAGGCGAGTGACTGGAGTTCAGACGTGTGCTCTTCCGATCT
30	ATAGCGTC	CAAGCAGAAGACGGCATACGAGATGACGCTATGTGACTGGAGTTCAGACGTGTGCTCTTCCGATCT
