# Supplementary material for: High-throughput barcoding of nanoparticles identifies cationic, degradable lipid-like materials for mRNA delivery to the lungs in female preclinical models
Source: Nat Commun. 2024 Feb 29;15:1884. doi: 10.1038/s41467-024-45422-9 (PMC10904786; doi:10.1038/s41467-024-45422-9)
Supplement: Supplementary file 3 — Description of Additional Supplementary Files [file 41467_2024_45422_MOESM3_ESM.pdf]

Description of Additional Supplementary Files

Supplementary Data Legends

Supplementary Data 1: Enrichment analysis for Figure 3e.

Supplementary Data 2: Library of b-DNA seuqnences. NNNNNNNNNN represents a unique molecular identifier (UMI). *denotes phosphorothioate modifications.

Supplementary Data 3: LC-MS spectrum of A2-6.

Supplementary Data 4: LC-MS spectrum of A2-6b.

Supplementary Data 5: LC-MS spectrum of A2-7.

Supplementary Data 6: LC-MS spectrum of A2-7b.

Supplementary Data 7: LC-MS spectrum of A2-7b2.

Supplementary Data 8: LC-MS spectrum of A2-8.

Supplementary Data 9: LC-MS spectrum of A2-8b.

Supplementary Data 10: LC-MS spectrum of A2-9.

Supplementary Data 11: LC-MS spectrum of A2-9b.

Supplementary Data 12: LC-MS spectrum of A2-9b2.

Supplementary Data 13: LC-MS spectrum of 5-A2-7b2.
