# Supplementary material for: High-throughput barcoding of nanoparticles identifies cationic, degradable lipid-like materials for mRNA delivery to the lungs in female preclinical models
Source: Nat Commun. 2024 Feb 29;15:1884. doi: 10.1038/s41467-024-45422-9 (PMC10904786; doi:10.1038/s41467-024-45422-9)
Supplement: Supplementary file 5 — Reporting Summary [file 41467_2024_45422_MOESM5_ESM.pdf]

nature portfolio

Corresponding author(s): Michael Mitchell

Last updated by author(s): Jan 10, 2024

Reporting Summary

Nature Portfolio wishes to improve the reproducibility of the work that we publish. This form provides structure for consistency and transparency in reporting. For further information on Nature Portfolio policies, see our Editorial Policies and the Editorial Policy Checklist.

Statistics

For all statistical analyses, confirm that the following items are present in the figure legend, table legend, main text, or Methods section.

n/a	Confirmed	
☐	☒	The exact sample size (*n*) for each experimental group/condition, given as a discrete number and unit of measurement
☐	☒	A statement on whether measurements were taken from distinct samples or whether the same sample was measured repeatedly
☐	☒	The statistical test(s) used AND whether they are one- or two-sided<br>*Only common tests should be described solely by name; describe more complex techniques in the Methods section.*
☐	☒	A description of all covariates tested
☐	☒	A description of any assumptions or corrections, such as tests of normality and adjustment for multiple comparisons
☐	☒	A full description of the statistical parameters including central tendency (e.g. means) or other basic estimates (e.g. regression coefficient) AND variation (e.g. standard deviation) or associated estimates of uncertainty (e.g. confidence intervals)
☐	☒	For null hypothesis testing, the test statistic (e.g. *F*, *t*, *r*) with confidence intervals, effect sizes, degrees of freedom and *P* value noted<br>*Give P values as exact values whenever suitable.*
☒	☐	For Bayesian analysis, information on the choice of priors and Markov chain Monte Carlo settings
☒	☐	For hierarchical and complex designs, identification of the appropriate level for tests and full reporting of outcomes
☒	☐	Estimates of effect sizes (e.g. Cohen's *d*, Pearson's *r*), indicating how they were calculated

Our web collection on statistics for biologists contains articles on many of the points above.

Software and code

Policy information about availability of computer code

Data collection	MestReNova 9.0, Flowjo V10, IVIS specturm, LAS X software (Leica)
Data analysis	Small animal imaging data were analyzed by the Living Image 4 Software (Caliper Life Sciences). Fluorescent microscopy imaging data were analyzed using LAS X software (Leica). All statistical calculations were performed using GraphPad Prism 9.0 (GraphPad Software Inc.) NMR and LC-MS spectra were analyzed using Mestre Nova 9.0 software (Mestre lab Research S.L.). Flow cytometry results were analyzed by FACS and FlowJo v10.

For manuscripts utilizing custom algorithms or software that are central to the research but not yet described in published literature, software must be made available to editors and reviewers. We strongly encourage code deposition in a community repository (e.g. GitHub). See the Nature Portfolio guidelines for submitting code & software for further information.

Data

Policy information about availability of data

All manuscripts must include a data availability statement. This statement should provide the following information, where applicable:

- Accession codes, unique identifiers, or web links for publicly available datasets
- A description of any restrictions on data availability
- For clinical datasets or third party data, please ensure that the statement adheres to our policy

All relevant data supporting the findings of this study are available within the paper and Supplementary Information. Source data are provided with this paper.

Human research participants

Policy information about studies involving human research participants and Sex and Gender in Research.

Reporting on sex and gender	N/A
Population characteristics	N/A
Recruitment	N/A
Ethics oversight	N/A

Note that full information on the approval of the study protocol must also be provided in the manuscript.

Field-specific reporting

Please select the one below that is the best fit for your research. If you are not sure, read the appropriate sections before making your selection.

☒ Life sciences ☐ Behavioural & social sciences ☐ Ecological, evolutionary & environmental sciences

For a reference copy of the document with all sections, see nature.com/documents/nr-reporting-summary-flat.pdf

Life sciences study design

All studies must disclose on these points even when the disclosure is negative.

Sample size	No effect size was predetermined, but sample sizes employed in this study are consistent with previously published works (Li A W, Sobral M C, Badrinath S, et al. Nature materials, 2018, 17(6): 528-534; or Kuai R, Ochyl L J, Bahjat K S, et al. Nature materials, 2017, 16(4): 489-496.). For example, in vitro studies were repeated at least three times independently and in the in vivo experiments with at least 3 mice per group were performed.
Data exclusions	No data was excluded from the analysis.
Replication	Experiments were repeated at least three independent experiments with similar results. All experiments were reproduced to reliably support conclusions stated in the manuscript.
Randomization	For in vivo FLuc and Cre mRNA delivery, cages of female mice were randomly selected and then divided into experimental groups for further in vivo dosing treatment. For the tumor model, tumor cells were inoculated into female mice aged 6-8 weeks of similar weight. On day 20 after tumor cell inoculation, mice were randomly assigned to four groups for therapeutic treatments.
Blinding	Investigators were blinded to group allocation during experiments. Investigators performing in vivo mRNA delivery and gene editing were blinded to saline and mRNA-LNPs treatment groups during data collection and analysis.

Reporting for specific materials, systems and methods

We require information from authors about some types of materials, experimental systems and methods used in many studies. Here, indicate whether each material, system or method listed is relevant to your study. If you are not sure if a list item applies to your research, read the appropriate section before selecting a response.

Materials & experimental systems

n/a	Involved in the study	
☐	☒	Antibodies
☐	☒	Eukaryotic cell lines
☒	☐	Palaeontology and archaeology
☐	☒	Animals and other organisms
☒	☐	Clinical data
☒	☐	Dual use research of concern

Methods

n/a	Involved in the study	
☒	☐	ChIP-seq
☐	☒	Flow cytometry
☒	☐	MRI-based neuroimaging

Antibodies

Antibodies used	BV421 anti-mouse CD45 (1:200, Cat#103134, Clone#30-F11), AF488 anti-mouse CD31 (1:200, Cat#102514, Clone#MEC13.3) and

Antibodies used	AF647 anti-mouse CD326 (EPCAM, 1:200, Cat#118212, Clone#G8.8) were purchased from BioLegend. Primary antibody used in this study was AF488 CD31 (1:200, BioLegend, Cat#102502, Cat#MEC13.3), goat anti-moue/rat CD31 (1:200, Cat#AF3628, R&D Systems), and rabbit anti-mouse GFP (1:200, Cat#ab183734, abcam). Secondary antibodies used in this study was AF488-conjugated donkey anti-rat (1:1000, Thermo Fisher Scientific, Cat#A-21208), AF488-conjuagted donkey anti-rabbit (1:1000, Cat#A-21206, Thermo Fisher Scientific), and AF555-conjugated donkey anti-goat (1:1000, Cat#A32816, Thermo Fisher Scientific).
Validation	All antibodies used in the study were commercial and validated by the manufactures. Species and application validations and citations for primary antibodies can be found from the manufacturer's websites.

Eukaryotic cell lines

Policy information about cell lines and Sex and Gender in Research

Cell line source(s)	HeLa cell were purchased from the American Type Culture Collection (ATCC), which were tested negative for mycoplasma in University of Pennsylvania cell center. GFP expressing Lewis Lung Carcinoma (LLC-GFP) cells were provided by Ellen Puré Laboratory (UPenn).
Authentication	A short tandem repeat DNA profiling method was used to authenticate the cell lines and the results were compared with reference database. There is no mycoplasma contamination in the above cell lines.
Mycoplasma contamination	Cell lines were tested for mycoplasma contamination. No mycoplasma contamination was found.
Commonly misidentified lines (See ICLAC register)	These cell lines we used were not listed in commonly misidentified lines in ICLAC Register.

Animals and other research organisms

Policy information about studies involving animals; ARRIVE guidelines recommended for reporting animal research, and Sex and Gender in Research

Laboratory animals	C57BL/6 mice (female, 6-8 weeks, 18-20g) and C57BL/6-Tg(CAG-EGFP)1Osb/J mice (female, 6-8 weeks, 18-20g) were ordered from Jackson laboratory and housed in a specific-pathogen-free animal facility at ambient temperature (22 ± 2 °C), air humidity 40%–70% and 12h dark/12h light cycle and had free access to water and chow (Cat#5053, LabDiet).
Wild animals	No wild animal was involved in this study.
Reporting on sex	Female mice were used in this study
Field-collected samples	The study did not involve samples collected from the field.
Ethics oversight	All animal experiment protocols were reviewed and approved by the institutional animal care and use committee of the University of Pennsylvania.

Note that full information on the approval of the study protocol must also be provided in the manuscript.

Flow Cytometry

Plots

Confirm that:

☒ The axis labels state the marker and fluorochrome used (e.g. CD4-FITC).

☒ The axis scales are clearly visible. Include numbers along axes only for bottom left plot of group (a 'group' is an analysis of identical markers).

☒ All plots are contour plots with outliers or pseudocolor plots.

☒ A numerical value for number of cells or percentage (with statistics) is provided.

Methodology

Sample preparation	Details of sample preparation are provided in the Methods section. Briefly, tissues samples were collected, cut into small pieces, and digested by DMEM medium containing collagen IV. The above cell suspension was then filtered, centrifuged, and lysed by ACK lysis buffer. Single-cell suspensions were obtained and stained with antibodies according to the manufacturer's protocols, and then analyzed by flow cytometry.
Instrument	Canto HiII (BD Biosciencs) and A3 lite (BD Biosciences)
Software	FACS Diva and FlowJo V10
Cell population abundance	The absolute cells around 100000 were analyzed for fluorescent intensity in the defined gate.

Gating strategy

Briefly, single cells were selected by FSC and SSC plots. Live cells were selected as defined by Live Dead Stain-negativity. Immune cells were gated by CD45+ cells. Endothelial cells were gated by CD45-/CD31+ cells. Epithelial cells were gated by CD45-/CD31-/ CD326+ cells. Other cells were gated by CD45-/CD31-/ CD326- cells. Detailed gating strategies were provided in the Supplementary Information Figure 25, 26, and 27.

☒ Tick this box to confirm that a figure exemplifying the gating strategy is provided in the Supplementary Information.
